# Inheritance and Variation of Cytosine Methylation in Three *Populus* Allotriploid Populations with Different Heterozygosity

**DOI:** 10.1371/journal.pone.0126491

**Published:** 2015-04-22

**Authors:** Yujing Suo, Chunbo Dong, Xiangyang Kang

**Affiliations:** 1 National Engineering Laboratory for Tree Breeding, College of Biological Sciences and Technology, Beijing Forestry University, Beijing, Peoples’ Republic of China; 2 Key Laboratory of Genetics and Breeding in Forest Trees and Ornamental Plants, Ministry of Education, College of Biological Sciences and Technology, Beijing Forestry University, Beijing, Peoples’ Republic of China; University of Gottingen, GERMANY

## Abstract

DNA methylation is an epigenetic mechanism with the potential to regulate gene expression and affect plant phenotypes. Both hybridization and genome doubling may affect the DNA methylation status of newly formed allopolyploid plants. Previous studies demonstrated that changes in cytosine methylation levels and patterns were different among individual hybrid plant, therefore, studies investigating the characteristics of variation in cytosine methylation status must be conducted at the population level to avoid sampling error. In the present study, an F1 hybrid diploid population and three allotriploid populations with different heterozygosity [originating from first-division restitution (FDR), second-division restitution (SDR), and post-meiotic restitution (PMR) 2n eggs of the same female parent] were used to investigate cytosine methylation inheritance and variation relative to their common parents using methylation-sensitive amplification polymorphism (MSAP). The variation in cytosine methylation in individuals in each population exhibited substantial differences, confirming the necessity of population epigenetics. The total methylation levels of the diploid population were significantly higher than in the parents, but those of the three allotriploid populations were significantly lower than in the parents, indicating that both hybridization and polyploidization contributed to cytosine methylation variation. The vast majority of methylated status could be inherited from the parents, and the average percentages of non-additive variation were 6.29, 3.27, 5.49 and 5.07% in the diploid, FDR, SDR and PMR progeny populations, respectively. This study lays a foundation for further research on population epigenetics in allopolyploids.

## Introduction

Polyploidization is a significant speciation mechanism for eukaryotes and is especially common in plants. More than 70% of flowering plants are polyploids [1, 2]. Interspecific hybridization and allopolyploidization occur frequently in various plant taxa, including *Brassica* [3], *Triticum* [4], *Spartina* [5], and *Senecio* [6]. Triploid breeding is a powerful approache for improvement of *Populus*. *Populus* triploids can be produced by crossing diploids with tetraploids, or utilization of spontaneous or artificial unreduced (2n) gametes. While considering the lack of tetraploid parents and weak competition of 2n pollen [7], hybridization with induced 2n eggs has been proven as an efficient method for triploid production [8]. Both the first and second phases of abnormal meiosis during megasporogenesis in plants, including first-division restitution (FDR) and second-division restitution (SDR), can contribute 2n gametes [9]. In addition, 2n eggs can also be derived from embryo sac chromosome doubling by post-meiotic restitution (PMR) [10].

Newly formed allopolyploids undergo a wide spectrum of genomic and epigenetic variation, causing phenotypic diversity. Previous studies suggested that epigenetic variability, rather than genetic variability, could explain the phenotypic variation in *Spartina* allopolyploids [11, 12]. Therefore, epigenetic mechanisms may play a significant role in the stabilization and evolution of allopolyploids. Of the inter-related epigenetic mechanisms that control gene expression, including histone covalent modification and small-interfering RNAs, DNA methylation variation in polyploid species has been one of the most widely investigated during the past decade. Several previous studies investigated cytosine methylation changes during the formation of allopolyploids. In *Arabidopsis* allotetraploids, 8.3% of the products scored showed cytosine methylaton changes, and 62.5% of the loci underwent demethylation [13]. The alterations in cytosine methylation occurred in ~13% of the loci, either in the F1 hybrid or in the wheat allopolyploid [4]. In contrast, 48% of the loci detected changes in the cytosine methylation status between parental genomes and the newly resynthesized *Brassica napus* allopolyploids, and de novo methylation occurred at a much higher frequency than de novo demethylation [14]. The findings of cytosine methylation variations in these studies were distinctly different. Moreover, changes in cytosine methylation levels and patterns could be directed or stochastic among individual hybrid plants [15]. Due to the potential effects of sampling error, further work at the population level is urgently needed to determine the significance of variation in cytosine methylation status [11].

In order to analyse the role of hybridization and polyploidization in cytosine methylation variation during the formation of allotriploids, we investigated the characteristics of inheritance and variation in cytosine methylation status in an F_1_ hybrid diploid population and three allotriploid populations with different heterozygosity relative to their common parents using methylation-sensitive amplification polymorphism (MSAP). This study lays a foundation for further research on population epigenetics in allopolyploids.

## Materials and Methods

### Plant materials

Floral branches of a female parent *Populus pseudo-simonii × P*. *nigra* ‘Zheyin3#’ (2n = 2x = 38, abbreviated as ZY3) were collected from a forest farm with the permission of Inner Mongolia Forestry Science Institute of Tongliao City (Inner Mongolia Autonomous Region, China). Floral branches of a male parent *P*. *beijingensis* (2n = 2x = 38, abbreviated as BJY) were collected at the Beijing Forestry University (Beijing, China). The branches were cultured in water to force floral development in a greenhouse.

### Artificial hybridization and chromosome doubling

According to the previous studies from our lab [8, 10], ZY3 buds were exposed to 41°C for 4 h at suitable stages during megasporogenesis to induce first-division restitution (FDR) and second-division restitution (SDR) 2n megaspores. When the stigmas of the ZY3-treated buds were receptive, they were pollinated with fresh BJY pollen. Moreover, to produce post-meiotic restitution (PMR) 2n eggs, some ZY3 buds were not treated (41°C for 4 h) until approximately 48–72 h of development after pollination (during which ZY3 catkins were in the embryo sac development stage, but not megasporogenesis stages for producing FDR and SDR 2n megaspores) [16]. Untreated buds were defined as the control group. After approximately 1 month of cultivation, the matured seeds were collected and germinated in sterile soil in the Beijing Forestry University greenhouse. When the seedlings had grown to a height of approximately 5 cm, they were transferred to containers with nutrient-rich soil to promote growth. Then the surviving seedlings were transplanted to the field. Using cuttage technique, branches of parents were also planted in the same field.

### Determination of the ploidy level

The ploidy level of all hybrids derived from the cross of ZY3 *×* BJY was determined by flow cytometric analysis. A known diploid plant derived from the same cross was used as a control. The diploid control was confirmed by somatic chromosome counting as described previously [17]. The ploidy level of the measured sample was determined by DNA content-associated peak shifts based on the diploid control. The flow cytometric analysis was carried out based on an established protocol [16]. Young and fresh leaves from the measured samples and the diploid control plant were chopped together using a sharp blade in a dish with 700 μl nuclei extraction solution (45 mM MgCl_2_, 30 mM sodium citrate, 20 mM morpholineopropane sulfonic acid, 1% (v/v) Triton X-100, pH 7.0). 40-μm nylon meshes were used as filter to remove large debris from the nuclear suspension. Next, the nuclei were stained with 100 μl 4’,6’-diamidino-2-phenylindole (DAPI, 5 μg/ml) for 30 s. Then, the prepared samples were measured by flow cytometry (CyFlow Ploidy Analyzer/Cy). Each putative triploid was assessed independently three times.

### Assessment the type of polyploidization

Six simple sequence repeat (SSR) markers with low recombination frequencies were used to assess the genetic composition of triploid hybrids derived from megaspore chromosome doubling by FDR and SDR. The protocol and results were described in the previous study from our lab [18]. The triploid hybrids derived from PMR 2n eggs were identified based on their treatment stage (48–72 h of development after pollination) during which ZY3 catkins were in the embryo sac development stage, but not megasporogenesis stages for producing FDR and SDR 2n megaspores [16]. For each population (FDR-, SDR-, PMR-triploid hybrids, normal diploid hybrids), 30 seedlings were selected randomly. Fully expanded leaves were collected and frozen immediately in liquid nitrogen and stored at—80°C.

### DNA extraction

Total genomic DNA was extracted from 300 mg of frozen leaves using a DNeasy Plant Mini Kit (Tiangen Biotech Co. Ltd, Beijing, China). DNA purity and concentration were determined using a NanoVue spectrophotometer (GE Healthcare China, Beijing, China).

### Methylation-sensitive amplification polymorphism (MSAP)

The methylation-sensitive amplification polymorphism (MSAP) technique is an efficient method for detecting alterations in cytosine methylation in plants, which is based on amplified fragment length polymorphism (AFLP). The isoschizomeric restriction enzymes *Hpa*II and *Msp*I were used in this study instead of *Mse*I. *Hpa*II and *Msp*I recognize the same restriction site (5’-CCGG-3’) but have different sensitivities to methylation of the cytosine residues. Specifically, *Hpa*II will not cut if either of the cytosine residues is full-methylated (both strands), whereas *Msp*I will not cut if the external cytosine is full-methylated or hemi-methylated (one strand) [19, 20]. Thus, different methylated status at the cytosines of the CCGG sites would be recognized by the different products obtained with *Eco*RI-*Hpa*II and *Eco*RI-*Msp*I.

MSAP analysis was performed as reported previously [21, 22], with some modifications. For each sample, equal amounts of DNA were digested using *Eco*RI/*Hpa*II or *Eco*RI/*Msp*I. After digestion, two different adapters (S1 Table) designed for the *Eco*RI and *Hpa*II/*Msp*I sticky ends were ligated to the DNA. The DNA fragments were diluted fivefold with sterile water for use as templates for pre-amplification with E_00_ and H/M_00_ primers (S1 Table). The pre-amplification products were then diluted tenfold for selective amplification. During the selective amplification, 27 pairs of *Eco*RI and *Hpa*II/ *Msp*I primers (S2 Table) containing two additional selective nucleotides were used for PCR amplification. The denatured PCR products were separated on a 6% polyacrylamide denaturing gel at 90 W for 2 h and visualized by silver staining. Only clear and reproducible bands that appeared in two independent PCR amplifications were scored.

### MSAP data analysis

The scored MSAP bands were transformed into a binary character matrix with scores of “1” for presence and “0” for absence of a band. The bands between 100 and 500 bp in length were recorded and classified into four types (S3 Table). The proportions of non-methylated, hemi-methylated, and full-methylated sites were calculated for 122 samples (parents, 30 samples × 4 hybrid progeny populations). All data were expressed as percentages and were subjected to a square root arcsine transformation process to normalize the distribution for each hybrid progeny population. Then the normalized percentages of methylated (hemi-, full-) sites of 120 hybrid progenies were processed using the SPSS software (SPSS for Windows, version13, SPSS, Chicago, IL) and One-Way ANOVA was used to analyze the cytosine methylation level differences in the four hybrid progeny populations. In addition, the inheritance and non-additive variation of cytosine methylation were analyzed.

## Results

**Fig 1:**
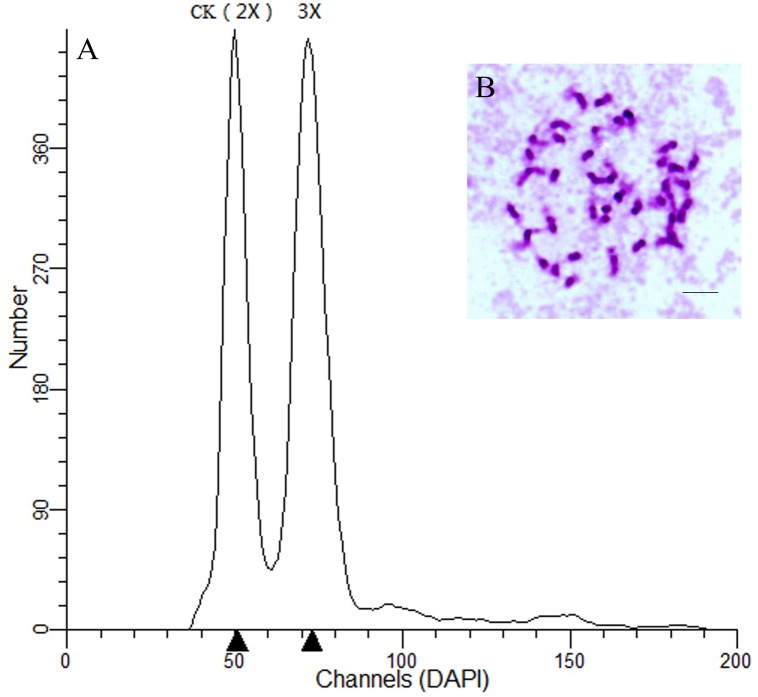
Result of ploidy level detection. (A) Flow cytometric analysis of nuclei mixtures from young leaves of the diploid control and triploid hybrid. (B) Somatic chromosome number in the diploid control (2n = 2x = 38), scale bar = 5 μm.

The ploidy levels of the hybrids were determined using flow cytometric analysis. The DNA content associated peak of each triploid sample was shifted to a position indicating 1.5 times the DNA content of the diploid control (Fig 1).

Using 27 pairs of *Eco*RI + *Hpa*II/*Msp*I selective primer combinations (S2 Table), we obtained 957 clear, reproducible bands for the Cathay poplar parents ZY3 and BJY and their four hybrid progeny populations. Based on the presence or absence of the bands, four band types were formed: Type I, bands present in both the *Hpa*II and *Msp*I digests (non-methylated); Type II, bands present in the *Hpa*II digest but absent from the corresponding *Msp*I digest (hemi-methylated); Type III, bands absent from the *Hpa*II digest but present in the *Msp*I digest (full-methylated); Type IV, bands absent from both the *Hpa*II and *Msp*I digests (full-methylated) (S3 Table). Based on the MSAP profiles, the number and frequency of non-methylated, hemi-methylated, and full-methylated CCGG sites were calculated.

### Cytosine methylation levels at CCGG sites in Cathay poplar parents and four hybrid progeny populations

**Fig 2:**
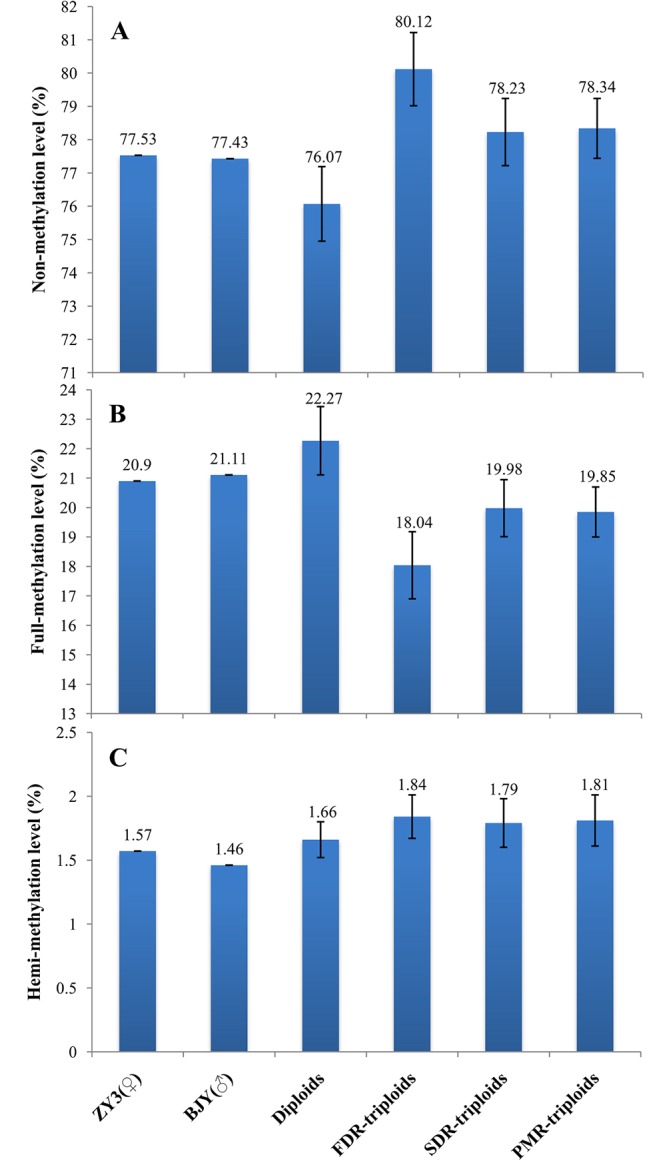
The average cytosine methylation levels in parents and their hybrid progeny populations. (A) Non-methylation level; (B) Full-methylation level; (C) Hemi-methylation level.

The levels of cytosine methylation at CCGG sites in the ZY3 and BJY Cathay poplar parental lines and their hybrid progeny populations with different ploidy levels and origins are presented in Fig 2. The total methylation levels in parents were 22.47% (215/957) in ZY3 and 22.57% (216/957) in BJY with hemi-methylation contributing 1.57 and 1.46%, respectively, and full-methylation contributing 20.90 and 21.11%, respectively. The maximum average total methylation level in the diploid hybrid progeny population was 23.93%, but was less in the FDR, SDR, and PMR triploid populations with values of 19.88, 21.77, and 21.66%, respectively (Fig 2), which indicated a negative correlation between the methylation levels and the ploidy levels. The full-methylation levels in the four hybrid progeny populations exhibited a trend similar to that of the total methylation level. However, the opposite was observed for the hemi-methylation levels, which increased markedly in all four hybrid progeny populations. Another notable result was that full-methylation was the major form of cytosine methylation in both the parents and their hybrid progenies (Fig 2). It is worth mentioning that type II (1–0, *Hpa*II-*Msp*I) MSAP bands pattern (S3 Table) is ambiguous [23]. It may be interpreted as two different situations: (i) hemi-methylated mCCGG sites; (ii) the presence of internal CmCGG site(s) between the cleaved distal CCGG and the *Eco*RI site. Therefore, the hemi-methylation level may be over-estimated.

Total methylation levels in the four hybrid progeny populations are shown in Table 1. The total methylation levels changed among individuals in each population. For the FDR triploid group, the total methylation levels ranged from 17.24 to 22.36% and exhibited the highest coefficient of variation (5.53%). The coefficients of variation for the diploid, SDR triploid, and PMR triploid populations were 4.68, 4.64, and 4.16%, respectively. The total methylation levels were significantly different among the hybrid progeny populations. Therefore, we used multiple comparisons for further analysis. The mean differences between the diploid population and three triploid populations were significant at the 0.05 level. The FDR triploid population had the lowest total methylation level, which was markedly different from the SDR and PMR triploid populations. However, the mean difference between the SDR and PMR triploid populations was 0.07646, indicating that the two populations were not significantly different in the total methylation level (S4 Table).

**Table 1:** Descriptives of the total cytosine methylation levels in the four hybrid progeny populations.

Groups	N	Mean	Std. Deviation	C.V(%)	Minimum	Maximum
Diploids	30	0.2393	0.0112	4.68	0.2090	0.2571
FDR-triploids	30	0.1988	0.0110	5.53	0.1724	0.2236
SDR-triploids	30	0.2177	0.0101	4.64	0.2048	0.2435
PMR-triploids	30	0.2166	0.0090	4.16	0.1933	0.2382

Note: Total cytosine methylation includes hemi-methylation and full-methylation.

### Inheritance and variation of cytosine methylation patterns in the four hybrid progeny populations

**Fig 3:**
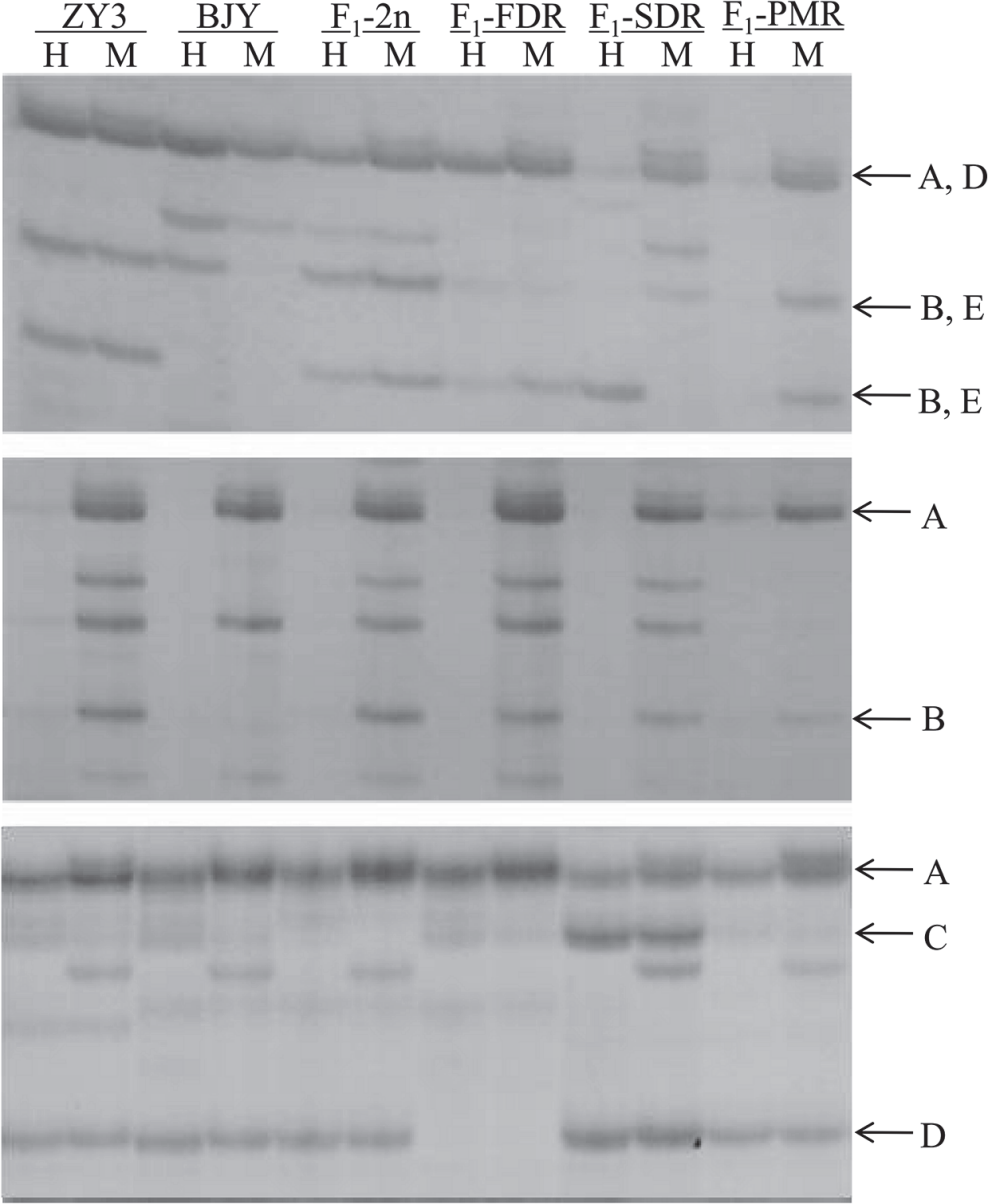
Examples of MSAP profiles showing the inheritance and variation of DNA methylation sites in F1 hybrids relative to their parental lines. H genomic DNA was digested with *Hpa*II and *Eco*RI; M genomic DNA was digested with *Msp*I and *Eco*RI; Primer combinations are E_00_+GC/ HM_00_+CT (a), E_00_+CC/HM_00_+TC (b), E_00_+GG/ HM_00_+TG (c). (A) Monomorphic inheritance; (B) Additivity inheritance; (C) Demethylated; (D) Hyper- methylated; (E) Unidentified types.

Based on the MSAP profile, inheritance and variation of cytosine methylation patterns occurred in the four hybrid progeny populations. As shown in Table 2, for inheritance, we observed the monomorphic and additivity parental patterns. For methylation variation, they were classified into three main classes, including hypermethylation, demethylation, and unidentified types (Fig 3).

**Table 2:** Inheritance and variation of cytosine methylation patterns.

Types		ZY3(♀)	BJY(♂)	F_1_-hybrids
		H M	H M	H M
Inheritance	Monomorphic	1 1	1 1	1 1
		0 1	0 1	0 1
		1 0	1 0	1 0
	Additivity	0 0	1 1	0 0
		1 1	0 0	1 1
		0 1	0 0	0 1
		1 0	0 0	1 0
		0 0	1 1	1 1
		1 1	0 0	0 0
		0 1	0 0	0 0
		1 0	0 0	0 0
Variation (non-additivity)	Demethylated	0 0	0 0	1 1
	Hyper-methylated	1 1	1 1	0 1
		1 1	1 1	0 0
	Unidentified	1 1	1 0	0 1
		1 1	1 0	0 0
		1 1	0 0	0 1
		1 1	0 0	1 0

Note: H. DNA were digested using *Eco*RI/*Hpa*II; M, DNA were digested using *Eco*RI/*Msp*I

We scored the cytosine methylation patterns of each hybrid separately. The statistical analysis of the inheritance and variation of methylation patterns in the four hybrid progeny populations is shown in Table 3. Of the 957 sites scored, 402 (42.01%) exhibited polymorphic methylation patterns between the parents and the hybrid progeny populations. A large majority (93.71–96.73%) of the polymorphic sites could be inherited from the parents and the frequency of the pattern variation was 3.27–6.29% (Table 3). These results showed that most cytosine methylation could be inherited from the parents to the progeny. For the four progeny populations, additivity of the methylated parental patterns was demonstrated in ~55% of the polymorphic sites. The FDR triploid population exhibited the lowest variation frequency (3.27%), which was markedly different from the other populations. However, the variation frequencies of the SDR and PMR triploid populations were not significantly different (S5 Table). In addition, hyper-methylation was the main variation type among the four hybrid progeny populations. Due to the limitations of the MSAP technique, unidentified variation types were present at a frequency of 0.41–0.80%.

**Table 3:** Inheritance and variation of cytosine methylation patterns in the four hybrid progeny populations.

Groups	Total polymorphic sites	Inheritance		Total	Variation			Total
		Monomorphic	Additivity		Demethylation	Hyper-methlyation	Unidentified	
Diploids	402	154.63^a^ (38.47^b^)	222.07 (55.24)	376.70 (93.71)	2.93 (0.73)	19.97 (4.97)	2.40 (0.60)	25.30 (6.29)
FDR-triploids	402	165.30 (41.12)	223.57 (55.61)	388.87 (96.73)	3.27 (0.81)	8.20 (2.04)	1.67(0.41)	13.13 (3.27)
SDR-triploids	402	158.80 (39.50)	221.13 (55.01)	379.93 (94.51)	4.40 (1.09)	14.47 (3.60)	3.20 (0.80)	22.07 (5.49)
PMR-triploids	402	159.60 (39.70)	222.00 (55.22)	381.60 (94.93)	3.87 (0.96)	14.43 (3.59)	2.10 (0.52)	20.40 (5.07)

Note: a, the number of corresponding sites; b, the frequency of corresponding sites (%).

## Discussion

Polyploids have genetic heterozygosity and phenotype characteristics that are closely related to parental selection and sexual polyploidization mechanisms. During the period of nucleus restructuring, the mechanisms of chromosome doubling for plant gametes are classified into three types: FDR, SDR, and PMR [24]. To our knowledge, research on cytosine methylation variation in polyploid populations generated by the three different reduplication mechanisms has been limited. Consequently, we explored the cytosine methylation variation in polyploid populations with different origins and heterozygosity in this study. By comparing the methylation patterns in diploid and allotriploid progenies with their original parents, the inheritance and variation of cytosine methylation accompanying hybridization and allopolyploidization could be brought to light.

### Differences among individual population members

Using 27 primer pairs and 30 samples for each hybrid population, we showed that the coefficients of variation for the total methylation level in the diploid, FDR, SDR, and PMR progeny populations were 4.68, 5.53, 4.64, and 4.16%, respectively (Table 1). These values demonstrate that the variation in cytosine methylation in each population differed substantially among individuals. Similar differences in methylation levels among individuals were reported in maize inbred lines [25], triploid asexual dandelion lineages [26], and synthetic *Arabidopsis* allotetraploids [13]. A reasonable explanation for this finding is that the stability of cytosine methylation at different genomic loci is variable [14, 15]. Some metastable loci show random alteration in some plants but not in others resulting in individual differences. Thus, the use of small sample sizes in cytosine methylation studies is likely to result in inaccuracy. To analyze the methylation variation more precisely during the formation of an allopolyploid, cytosine methylation must be investigated at the population level.

### Cytosine methylation level and pattern variation

Based on genome-wide marker data, we showed that the total methylation levels of the three triploid populations were significantly lower than those of the parents and the diploid population, and that the main methylation type was full-methylation (Fig 2). In addition, a large majority of the methylation patterns could be stably inherited by the F_1_ hybrids from the parents, and hyper-methylation was the main contributor to variation of methylation patterns (Table 3). An average of 3.27% of the polymorphic sites in the FDR triploid population changed their cytosine methylation patterns, which was less than in the diploid population (6.29%). Consequently, the probability of hypermethylation variation in the FDR triploid population was less than that in the diploid population, ultimately resulting in a lower methylation level in the FDR triploid population. Compared to diploid hybrids, the lower methylation level in triploid hybrids might be a readjusting reaction to the increase in ploidy for the sake of genomic stability.

Changes in cytosine methylation levels and patterns during the process of hybridization and polyploidization have been well documented. When comparing our results to other studies, we found that the variations in methylation levels observed in polyploid watermelon (*Citrullus lanatus*) [27], *Salvia* [28], and *Cucumis* [29] were similar to our findings (*i*.*e*., the methylation level of the triploid was less than the diploid). Conversely, Chen CB *et al*. surveyed the cytosine methylation variations in triploid black poplar and white poplar using MSAP and found that the total methylation level of poplar increased with increased ploidy [30]. We speculate that the inconsistent conclusions in these studies likely resulted from the small sample size (five samples for each species).

By comparing the methylation patterns of diploid and triploid hybrid populations with their parents, we showed that the vast majority of methylation patterns could be inherited from the parents, and that the average percentages of methylation pattern variation were 6.29, 3.27, 5.49, and 5.07% in the diploid, FDR, SDR, and PMR progeny populations, respectively (Table 3). This finding is similar to the results for allopolyploid C*ucumis* [29]. In addition, the main type of methylation pattern variation was hypermethylation, which is consistent with the results obtained for a synthesized wheat allotetraploid [31], triploid asexual dandelion [26], and *Brassica napus* allopolyploids [14]. Therefore, we suppose novel cytosine methylation occur in some sites during the formation of allotriploid, as a relatively stable repressive epigenetic regulator, protecting genome stability from the double shock of heterozygosity and polyploidy. However, in some other species, such as allotetraploid *Arabidopsis* [13], triploid *Citrullus lanatus* [27], and allopolyploid *Raphanobrassica* [32], hypomethylation or demethylation were more frequent than hypermethylation. This difference in methylation pattern variation suggests that cytosine methylation variation acts as an intricate regulatory mechanism responding to hybridization and polyploidization.

### Cytosine methylation variation resulting from hybridization and/or genome duplication

Whether cytosine methylation variation is induced by hybridization or genome duplication in polyploid plants is controversial. Madlung A *et al*. [13] and Salmon A *et al*. [11] claimed that changes in methylation most likely result from hybridization rather than from changes in ploidy. However, Hegarty MJ *et al*. [33] investigated the non-additive changes of cytosine methylation in allopolyploid *Senecio* and showed that, despite of the significant effect of interspecific hybridization, polyploidization led to a secondary effect on methylation with reversion to additivity at some loci and novel methylation at others. Similarly, we found that 6.29% of loci exhibited cytosine methylation variation in the F_1_ hybrid diploid population (Table 3) largely as the result of interspecific hybridization. Nevertheless, the methylation changes showed different levels of reversion in the three triploid populations, particularly in the FDR triploid population (3.27%), approximately half that of the diploid population, indicating that polyploidization also has a significant effect on methylation.

A majority of cytosine methylation can be inherited from parents and a small but significant proportion of loci show non-additive changes in hybrid diploid and triploid populations resulting from both hybridization and polyploidization. To deepen our understanding of the role of DNA methylation variation in the formation and evolution of allopolyploidy, further work focusing on DNA methylation variation in the key genome regions related to phenotypic variation and the mechanism of epigenetic regulation of gene expression is needed. The present study lays a foundation for further research on population epigenetics in allopolyploids.

## Supporting Information

S1 Table Adapter and primer sequences. (DOCX)

S2 Table Primer combinations used for MSAP analysis. (DOCX)

S3 Table The methylation sensitivity and band types of *Eco*RI and *Hpa*II/ *Msp*I. (DOCX)

S4 Table Multiple Comparisons of the normalized total cytosine methylation levels in the four hybrid progeny populations. (DOCX)

S5 Table Multiple Comparisons of the normalized cytosine methylation patterns variation in the four hybrid progeny populations. (DOCX)
