# Supplementary material for: Inheritance and Variation of Cytosine Methylation in Three Populus Allotriploid Populations with Different Heterozygosity
Source: PLoS One. 2015 Apr 22;10(4):e0126491. doi: 10.1371/journal.pone.0126491 (PMC4406749; doi:10.1371/journal.pone.0126491)
Supplement: S1 Table — (DOCX) [file pone.0126491.s001.docx]

**S1 Table. Adapter and primer sequences.**

|  | *Eco*RI (5’-3’) | *Hpa*II/ *Msp*I (5’-3’) |
| --- | --- | --- |
| Adapter 1 | CTCGTAGACTGCGTACC | GACGATGAGTCTCGAT |
| Adapter 2 | AATTGGTACGCAGTCTAC | CGATCGAGACTCAT |
| Pre-amp primer | GACTGCGTACCAATTCA (E_00_) | ATGAGTCTCGATCGG (HM_00_) |
| Selective primer | GACTGCGTACCAATTCAAC (E1) | ATGAGTCTCGATCGGAT (HM1) |
|  | GACTGCGTACCAATTCACC (E2) | ATGAGTCTCGATCGGAG (HM2) |
|  | GACTGCGTACCAATTCAAG (E3) | ATGAGTCTCGATCGGTC (HM3) |
|  | GACTGCGTACCAATTCACG (E4) | ATGAGTCTCGATCGGTG (HM4) |
|  | GACTGCGTACCAATTCACA (E5) | ATGAGTCTCGATCGGCA (HM5) |
|  | GACTGCGTACCAATTCAGC (E6) | ATGAGTCTCGATCGGCT (HM6) |
|  | GACTGCGTACCAATTCACT (E7) | ATGAGTCTCGATCGGGA (HM7) |
|  | GACTGCGTACCAATTCAGG (E8) | ATGAGTCTCGATCGGTT (HM8) |
|  |  | ATGAGTCTCGATCGGAC (HM9) |
|  |  | ATGAGTCTCGATCGGTA (HM10) |
|  |  | ATGAGTCTCGATCGGAA (HM11) |
