# Supplementary material for: Inheritance and Variation of Cytosine Methylation in Three Populus Allotriploid Populations with Different Heterozygosity
Source: PLoS One. 2015 Apr 22;10(4):e0126491. doi: 10.1371/journal.pone.0126491 (PMC4406749; doi:10.1371/journal.pone.0126491)
Supplement: S2 Table — (DOCX) [file pone.0126491.s002.docx]

**S2 Table. Primer combinations used for MSAP analysis**

|  | E1 | E2 | E3 | E4 | E5 | E6 | E7 | E8 |
| --- | --- | --- | --- | --- | --- | --- | --- | --- |
| HM1 |  |  |  | **√** | **√** |  | **√** | **√** |
| HM2 | **√** |  |  |  |  |  |  |  |
| HM3 | **√** |  |  |  |  | **√** |  | **√** |
| HM4 |  | **√** |  |  |  |  | **√** | **√** |
| HM5 | √ |  |  |  |  | **√** |  | **√** |
| HM6 |  | **√** |  |  |  |  |  |  |
| HM7 |  | **√** |  |  |  |  |  |  |
| HM8 | **√** |  |  |  |  |  | **√** |  |
| HM9 | **√** |  |  |  | **√** |  | **√** |  |
| HM10 |  |  | **√** |  |  |  | **√** |  |
| HM11 |  |  | **√** |  | **√** | **√** |  | **√** |
