# Supplementary material for: Inheritance and Variation of Cytosine Methylation in Three Populus Allotriploid Populations with Different Heterozygosity
Source: PLoS One. 2015 Apr 22;10(4):e0126491. doi: 10.1371/journal.pone.0126491 (PMC4406749; doi:10.1371/journal.pone.0126491)
Supplement: S3 Table — (DOCX) [file pone.0126491.s003.docx]

**Table S3.** **The methylation sensitivity and band types of *Eco*RI and *Hpa*II/ *Msp*I**

| Type | *E*+*H* | *E*+*M* | State of CCGG site | Methylation state of CCGG site |
| --- | --- | --- | --- | --- |
| I | 1 | 1 | CCGG/GGCC | Non-methylated |
| II | 1 | 0 | ^m^CCGG/GGCC | Single-stranded external cytosine methylated |
| III | 0 | 1 | C^m^CGG/GG^m^CC | Double-stranded internal cytosine methylated |
| IV | 0 | 0 | ⒈^m^C^m^CGG/GG^m^C^m^C | Double-stranded internal & external cytosine methylated |
|  |  |  | ⒉^m^CCGG/GGC^m^C | Double-stranded external cytosine methylated |
