# Supplementary material for: Inheritance and Variation of Cytosine Methylation in Three Populus Allotriploid Populations with Different Heterozygosity
Source: PLoS One. 2015 Apr 22;10(4):e0126491. doi: 10.1371/journal.pone.0126491 (PMC4406749; doi:10.1371/journal.pone.0126491)
Supplement: S4 Table — (DOCX) [file pone.0126491.s004.docx]

**S4 Table. Multiple Comparisons of the normalized total cytosine methylation levels in the four hybrid progeny populations.**

| (I) Group | (J) Group | Mean Difference (I-J) | Std. Error | Sig. |
| --- | --- | --- | --- | --- |
| Diploids | FDR-triploids | 2.80566* | .18551 | .000 |
|  | SDR-triploids | 1.47108* | .18551 | .000 |
|  | PMR-triploids | 1.54755* | .18551 | .000 |
| FDR-triploids | Diploids | -2.80566* | .18551 | .000 |
|  | SDR-triploids | -1.33457* | .18551 | .000 |
|  | PMR-triploids | -1.25811* | .18551 | .000 |
| SDR-triploids | Diploids | -1.47108* | .18551 | .000 |
|  | FDR-triploids | 1.33457* | .18551 | .000 |
|  | PMR-triploids | .07646 | .18551 | .681 |
| PMR-triploids | Diploids | -1.54755* | .18551 | .000 |
|  | FDR-triploids | 1.25811* | .18551 | .000 |
|  | SDR-triploids | -.07646 | .18551 | .681 |

Note: *****. The mean difference is significant at the 0.05 level.
