# Supplementary material for: Inheritance and Variation of Cytosine Methylation in Three Populus Allotriploid Populations with Different Heterozygosity
Source: PLoS One. 2015 Apr 22;10(4):e0126491. doi: 10.1371/journal.pone.0126491 (PMC4406749; doi:10.1371/journal.pone.0126491)
Supplement: S5 Table — (DOCX) [file pone.0126491.s005.docx]

**S5 Table. Multiple Comparisons of the normalized cytosine methylation patterns variation in the four hybrid progeny populations.**

| (I) Group | (J) Group | Mean Difference (I-J) | Std. Error | Sig. |
| --- | --- | --- | --- | --- |
| Diploids | FDR-triploids | 4.23439* | .41659 | .000 |
|  | SDR-triploids | 1.00683* | .41659 | .017 |
|  | PMR-triploids | 1.52002* | .41659 | .000 |
| FDR-triploids | Diploids | -4.23439* | .41659 | .000 |
|  | SDR-triploids | -3.22757* | .41659 | .000 |
|  | PMR-triploids | -2.71438* | .41659 | .000 |
| SDR-triploids | Diploids | -1.00683* | .41659 | .017 |
|  | FDR-triploids | 3.22757* | .41659 | .000 |
|  | PMR-triploids | .51319 | .41659 | .220 |
| PMR-triploids | Diploids | -1.52002* | .41659 | .000 |
|  | FDR-triploids | 2.71438* | .41659 | .000 |
|  | SDR-triploids | -.51319 | .41659 | .220 |

Note: *****. The mean difference is significant at the 0.05 level.
